# Uniform criteria for total hip replacement surgery in patients with hip osteoarthritis: a decision tool to guide treatment decisions

**DOI:** 10.1093/intqhc/mzab030

**Published:** 2021-02-22

**Authors:** Femke Atsma, Olivier Molenkamp, Heinse Bouma, Stefan B Bolder, A Stef Groenewoud, Gert P Westert

**Affiliations:** Radboud University Medical Center, Radboud Institute for Health Sciences, Scientific Center for Quality of Healthcare, Geert Grooteplein Noord 21, P.O. Box 9101, 6500 HB Nijmegen, The Netherlands; Quin, Willem Fenengastraat 17, 1096 BL, Amsterdam, The Netherlands; Department of Orthopedic Surgery, Bergman Clinics, Rijksweg 69, 1411 GE, Naarden, null, The Netherlands; Department of Orthopedic Surgery, Amphia Hospital, Molengracht 21, 4818 CK, Breda, The Netherlands; Radboud University Medical Center, Radboud Institute for Health Sciences, Scientific Center for Quality of Healthcare, Geert Grooteplein Noord 21, P.O. Box 9101, 6500 HB Nijmegen, The Netherlands; Radboud University Medical Center, Radboud Institute for Health Sciences, Scientific Center for Quality of Healthcare, Geert Grooteplein Noord 21, P.O. Box 9101, 6500 HB Nijmegen, The Netherlands

**Keywords:** quality improvement, appropriateness, underuse and overuse, shared decision-making, hospital care

## Abstract

**Background:**

Uniform criteria for performing hip replacement surgery in hip osteoarthritis patients are currently lacking. As a result, variation in surgery and inappropriateness of care may occur. The aim of this study was to develop a consensus-based decision tool to support the decision-making process for hip replacement surgery.

**Methods:**

Patients with a diagnosis of unilateral or bilateral osteoarthritis were included. Consensus rounds with orthopedic surgeons were organized to blindly reassess medical files and to decide whether surgery is indicated or not, based on all available pre-treatment information. We compared the outcomes obtained from the blind reassessment by the consensus group with the actual treatment. Furthermore, prediction models were fitted on the reassessment outcome to identify which set of clinical parameters would be most predictive and uniformly shared in the decision to operate.

Two prediction models were fitted, one model without radiologic outcomes and one model where radiologic outcomes were included.

**Results:**

In total, 364 medical files of osteoarthritis patients were included and reassessed in the analyses. Key predictors in the prediction model without radiology were age, flexion, internal rotation and the Hip disability and Osteoarthritis Outcome Score–quality of life. The discriminative power was high (Area Under Receiver Operating Curve (AUC) = 0.86). Key predictors in the prediction model with radiology were age, internal rotation and Kellgren and Lawrence severity score (AUC = 0.94).

**Conclusion:**

The study yielded a decision tool with uniform criteria for hip replacement surgery in osteoarthritis patients. The tool will guide the clinical decision-making process of physicians on whether to perform hip surgery and should be used together with information about patient preferences and social context.

## Introduction

The number of total hip replacement surgeries per 100 000 inhabitants has substantially increased over the past years worldwide [1]. Overall, the rate of hip replacements increased by 30% between 2007 and 2017. In addition, a substantial variation between countries has been reported. In Western Europe, in 2017, utilization rates varied from 91 per 100 000 inhabitants in Portugal to 309 per 100 000 inhabitants in Germany, which comes down to an international variation factor of 3.4. A utilization rate of 238 per 100 000 inhabitants was reported for the Netherlands, which places it in the top 10 countries with the highest degree of hip replacement surgeries worldwide [1].

Possible explanations for this increase over time and geographical differences include variation in classification systems and variation in registration practices. Registrations may have improved over time and differences between countries may occur as a result of diagnosis definition. In case of hip replacement surgeries, some countries include both total and partial hip replacements while other countries only include total hip replacements [1]. Even within countries, significant variation in healthcare utilization exists. Diffuse clinical criteria for certain procedures may be important drivers for geographical variation in the over- and underuse of this procedure [2–4]. This may also be the case for hip replacement surgery, since clinical criteria for total hip replacements are based on limited and low-quality evidence [5]. Several other studies have indicated that there is an important variability in clinical criteria for hip replacements and that decision support tools, to decide which clinical parameters are most indicative of surgery, are imperative [6–8]. In order to reduce unwarranted practice variation and possible over- and underuse of hip replacement surgery, it is crucial to develop indication standards with clear and uniform clinical criteria for hip replacement surgery. In this way, the phenomenon of over- and underuse and subsequent practice variation will be addressed beforehand, namely when patients present themselves with symptoms of osteoarthritis, rather than having to conclude afterward that considerable variation existed, without knowing what part of the variation was unwarranted and what part of health care was inappropriate.

Most studies on this topic were aimed at improving explicit criteria for hip replacement surgery by evaluating outcomes after surgery [9–12]. Besides, most studies did not include radiologic severity in the models [13]. We state however that evaluating criteria in the total group of patients, including those not receiving surgery, is crucial when the aim is to define uniform clinical criteria for hip surgery and to identify key clinical parameters in the decision-making process. Uniform criteria for hip surgery should be clear in order to improve and facilitate decision-making by the orthopedic surgeon. To reach final treatment decisions, patient preferences and social context should be taken into account and added to the objective clinical criteria. The current paper focuses on objectifying these clinical criteria.

This project therefore aims to define uniform clinical criteria for the appropriateness of hip replacement surgery in a cohort of surgical and non-surgical hip osteoarthritis patients. We developed a consensus-based decision tool to identify uniform clinical parameters of hip replacement surgery in order to facilitate the decision-making process.

## Methods

### Design and study population

The study was designed as an observational study with longitudinal, prospectively collected data from medical files. We included patients with a diagnosis of unilateral or bilateral osteoarthritis who were treated at the orthopedic departments of two hospitals in the Netherlands (between 1 September 2016 and 30 June 2017). All patients were over 18 years of age at the time of diagnosis. Treatment consisted of either a total hip replacement or conservative treatment consisting of physiotherapy, intra-articular injection, medication and/ or lifestyle adjustments. We excluded patients with hip complaints related to implant problems rather than osteoarthritis, which would require a revision procedure.

The methodology used in this study adheres to the TRIPOD guideline for multivariable prediction models (TRIPOD: Transparent reporting of a multivariable prediction model for individual prognosis or diagnosis) [14].

### Data collection

We collected data from a period of 3 months before treatment. All data, except for patient-reported outcome measures (PROMs), were retrieved from electronic hospital medical records. PROMs were obtained by self-administered questionnaires.

We collected information with respect to patient characteristics, physical examination, radiology, PROMs and quality of life. Patient characteristics included age, sex, BMI (kg/m^2^), smoking and information on co-morbidities from other medical specialties. Physical examination was performed when the patient was lying in a supine position (except for the Trendelenburg walking pattern) and included the following clinical parameters: Trendelenburg walking pattern, Drehmann sign, flexion, extension, internal and external rotation, abduction and adduction. With respect to radiology, we used the Kellgren and Lawrence classification to grade the radiological severity of hip osteoarthritis based on a standard X-ray of the pelvis [15]. This composite score is based on cartilage loss, osteophytes, sclerosis and observed deformities. The score ranges from no abnormalities (a score of 0) to severe abnormalities (a score of 4). We were also able to use the separate radiological parameters, underlying the Kellgren and Lawrence score. We collected PROMs and quality of life through a self-administered questionnaire at baseline. The patients were asked to fill out the Hip disability and Osteoarthritis Outcome Score (HOOS) [16], resulting in five subscales (HOOS_symptoms&stiffness, HOOS_pain, HOOS_daily living, HOOS_activities&sports and HOOS_quality of life) [16], the EuroQol 5D (EQ-5D) [17] and the Oxford Hip Score [18].

### Blinded review of medical records

For the decision tool, we constructed a prediction model to identify clinical parameters that would be most decisive for surgery. We developed this model on a consensus-based clinical decision to either perform or not perform a total hip replacement. For that purpose, we organized an expert panel of orthopedic surgeons to reach a consensus about the indication to operate. We asked seven orthopedic surgeons from the two hospitals in this study (three and four surgeons per hospital) to blindly reassess the medical files of the included patients and to reach a consensus about the treatment. All clinical information from medical records was available to the orthopedic surgeons, including patient characteristics, information on the regime of conservative treatments (physiotherapy, pain treatment and injections) and comorbidity. Surgeons were blinded with respect to the actual treatment, because this was the main outcome of the blinded review. The consensus procedure resulted in the following output: (I) consensus reached, surgery; (ii) consensus reached, no surgery and (III) no consensus reached, surgery uncertain.

### Statistical analyses

First, we performed descriptive analyses to explore the data, investigate missing values and describe the characteristics of the patient population. To compare the blind assessment with the actual performance of surgery, we constructed a cross tabulation of the consensus output (surgery indicated/surgery not indicated) with the treatment that was actually performed (surgery performed/conservative treatment).

Second, we constructed the prediction models. We performed ordinary logistic regression analyses, using the reassessment from the consensus procedure as outcome variable (surgery versus no surgery) and patient-related information as well as clinical pre-treatment information as candidate predictors of the outcome. First, we performed bivariate analyses between the outcome and individual predictors. Predictors with a *P*-value > 0.15 were included in a multivariable model. The multivariable model was reduced by manually removing candidate predictors with a *P*-value > 0.15 based on the log likelihood ratio test. We also used a backward selection procedure to verify whether the same variables were selected. For continuous predictors, the linearity assumption was tested by including quadratic and tertiary terms in the models.

As a result, two models containing predictors that gained maximum discriminative power were constructed. The first model was based on information related to patient characteristics, physical examination, patient-reported outcomes and quality of life (HOOS, EQ-5D). The second model was based on the same information and radiology. We added the predictive value of the radiologic Kellgren and Lawrence classification to this model. For both models, intercept and regression coefficients with standard errors were presented, which can be used to calculate predictions. The predictive accuracy of the prediction models was estimated by calibration (goodness of fit and Hosmer and Lemeshow method) and discrimination properties (Area Under the Receiver Operating Curve, AUC) [19, 20].

Finally, bootstrapping techniques were performed to internally validate the final models and to adjust the estimated model performance for highly optimistic predictions. Random bootstrap samples were drawn with replacement from the derivation set consisting of all patients (1000 replications). Based on the performance of the models in the bootstrap samples, the coefficients from the prediction models and the AUC's were adjusted.

All analyses were performed with R version 3.4.0.

## Results

We included a total of 394 medical files from osteoarthritis patients in our study. In 70% of these files, immediate consensus was reached in the first online round. The remaining 30% of the files were discussed in the subsequent meeting with orthopedic surgeons to reach the final consensus. In 30 medical files, no consensus was reached on the outcome in the blinded evaluation procedure (7.6% of total). These patients were excluded, which left 364 medical files for the analyses. Table 1 presents the baseline characteristics of the study population. Patients were mainly female (61.5%) and had a mean age of 62.1 years (SD: 11.0). Patients receiving surgery (51.4%) or conservative (48.6%) treatment were almost equally divided in the research group.

**Table 1:** Baseline information from 364 osteoarthritis patient files

	*N*	Mean (SD)
Age (years)	364	62.1 (11.0)
BMI (kg/m^2^)	268	26.7 (3.8)
*Missing values*	*96 (26.4%)*	
		%
Sex		
Men	224	61.5
Women	140	38.5
Smoking		
Yes	295	80.8
No	45	12.4
*Missing values*	*24*	*6.8*
Co-morbidity		
Yes	251	69.0
No	88	24.2
*Missing values*	*25*	*6.8*
Hip replacement surgery		
Yes	187	51.4
No	177	48.6
Bilateralism		
Unique patients	326	89.6
Duplicate patients (bilateral treatment)	38	10.4

In Table 2, we compared the outcome of the blinded consensus procedure with the treatment that was actually performed. In 9.6% of the cases, surgery was indicated by the group of experts in the consensus procedure based on clinical parameters, while in reality no surgery had been performed. In contrast, in 13.5% of the cases no surgery was indicated by the group of experts, while in reality surgery had actually been performed.

**Table 2:** Comparison between the outcome of the blinded consensus procedure and the actual treatment of choice

	No surgery was performed	Surgery was performed	Total
Blinded consensus procedure: No surgery indicated	142 (39.0%)	49 (13.5%)	191
Blinded consensus procedure: Surgery indicated	35 (9.6%)	138 (37.9%)	173
Total	177 (48.6%)	187 (51.4%)	364

**Figure 1:**
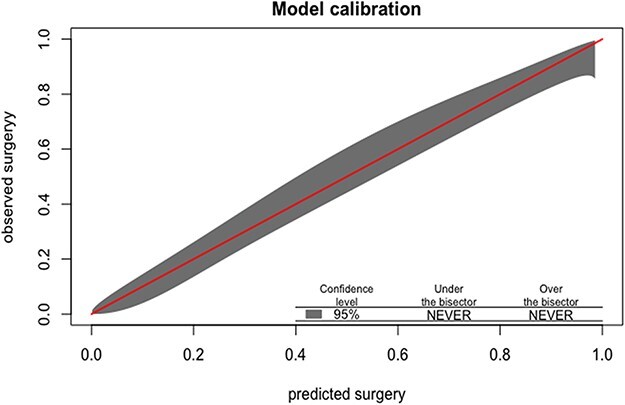
Calibration curve of the model without radiologic parameters.

**Figure 2:**
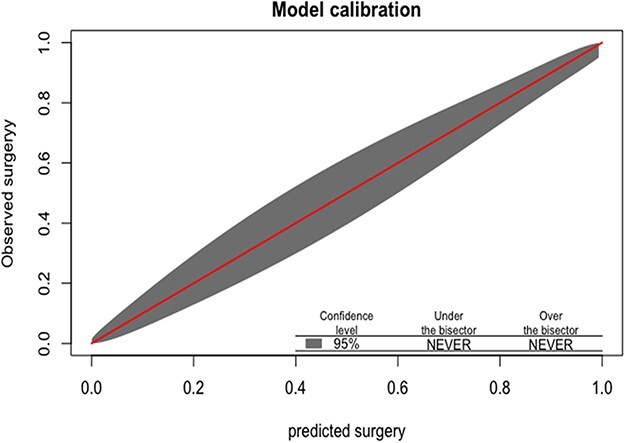
Calibration curve of the model with radiologic predictors.

Table 3 shows the prediction models. Model 1 pertains to the model without radiology, and Model 2 to the model with radiology in terms of the Kellgren and Lawrence classification. Model 1 had a very good predictive value for the consensus-based surgery with an AUC of 0.86 after internal validation. Predictors for hip replacement surgery were increased age, decreased flexion, endorotation > 0 and decreased HOOS_quality of life. When we added radiologic parameters, the model performance further increased to an AUC of 0.94 after internal validation. In this model, the factors flexion and HOOS_quality of life did not have any added predictive value. Calibration plots of both models were good with non-significant Hosmer and Lemeshow tests (Figures 1 and 2).

**Table 3:** Prediction models hip replacement surgery

	Model 1 (without radiology)		Model 2 (with radiology)	
*N* in model	364	364	364	364
AUC	0.87	0.86	0.94	0.94
	β (SE^a^)	β_*adjusted*^b^	β (SE^a^)	β_*adjusted*^b^
Intercept	−6.55 (1.63)	−5.91	−5.35 (1.35)	−4.79
Age (per year)	0.07 (0.02)	0.06	0.07 (0.02)	0.06
Flexion			*No predictor in this model*	*No predictor in this model*
≤90	4.23 (1.08)	3.81		
91–110	2.71 (1.05)	2.43		
111–120	1.84 (1.06)	1.66		
>120	Ref^c^	Ref		
Internal rotation				
0	Ref	Ref	Ref	Ref
>0	−1.84 (0.34)	−1.65	−1.97 (0.41)	−1.76
HOOS_quality of life			*No predictor in this model*	*No predictor in this model*
≤30	1.38 (0.74)	1.25		
31–40	0.60 (0.72)	0.54		
41–50	0.42 (0.77)	0.38		
>50	Ref	Ref		
Kellgren and Lawrence				
0–2	*NA*	*NA*	Ref	Ref
>2			3.93 (0.39)	3.53

a SE, standard error.

b Adjusted for overfitting after bootstrapping with uniform shrinkage.

c Ref, reference category.

In a sensitivity analysis, we implemented a modeling strategy to investigate the influence of the 30 patients for whom no consensus could be reached in de-blinded review. We included these 30 medical cases first as ‘surgery indicated’ in the model and thereafter as ‘no surgery indicated’ in the model. The results did not change in either approach, confirming that the models were very robust.

## Discussion

### Statement of principal finding

In this study, we developed consensus-based decision tools to identify the clinical parameters that are uniform and commonly shared in the decision-making process for surgery in hip osteoarthritis. These tools may enhance and support the decision to perform a total hip surgery in osteoarthritis patients or to follow a conservative treatment strategy. In this study, we constructed two prediction models with high predictive performances—one model without radiologic parameters and one model with radiologic parameters. The decision tools will clarify and objectify the decision-making process in the treatment of hip osteoarthritis patients.

### Strengths and limitations

Strengths of this study are the completeness of data and having variables without many missing values. Only for BMI the number of missing values was substantial, while it was moderate for smoking and co-morbidity. However, BMI, smoking and co-morbidity could be left out of the final model since they did not show high predictive properties in the univariate analyses.

A limitation of the study was the fact that the model was developed for a particular group of patients in two general hospitals. We acknowledge that, like all newly developed prediction models, the models may perform differently in another group of surgeons and patients. Therefore, it is important to validate the models in another setting with other groups of osteoarthritis patients and orthopedic surgeons. After this external validation, the models should be implemented as applicable tools in clinical practice.

### Interpretation in context of wider literature

In the past, several studies have been conducted to improve and objectify criteria for hip replacement surgery in osteoarthritis patients. For instance, the models developed by Quintana and colleagues are most widespread and validated models [21–23]. The researchers investigated the appropriateness of total hip replacement surgery by judging all possible combinations of clinical variables (216 scenarios) [21, 22]. The authors estimated the proportion of inappropriate care at 13.6%, which is in line with the estimated proportion of 13.5% for non-indicated surgery that we found. Differences between the group decision and the actual treatment may be explained by over- and under-treatment. However, we must be careful with drawing this conclusion; the discrepancy between the consensus-based indication and the real treatment in our study could also result from the effect of patient preferences. Even though the focus of the study is on objective clinical criteria, these patient preferences do play a role in final treatment decisions. Drawbacks of the Quintana models were the omission of radiologic severity in the models and the high proportion of scenarios classified as uncertain by the expert panel (46% in the 2000 study and 21% in the 2005 study). These drawbacks were also pointed out by Dowsey and colleagues in a review on the tools available for assistance in assessing the appropriateness for total joint replacement [13]. Dowsey and colleagues stressed the importance of taking into account radiologic severity in studies on the appropriateness of total hip replacement, since literature has shown radiologic severity to be an important potential predictor for total hip replacement in both the short and the long terms [13, 24, 25]. Additionally, there is literature on the predictive value of pre-operative information for outcomes after total hip replacement in patients with arthritis. However, these studies only included patients with osteoarthritis undergoing hip replacement surgery and did not investigate the natural course of symptoms in patients not receiving hip surgery [9–11, 23, 26]. In contrast, we included both groups (surgical and conservatively treated patients) to identify indicators that either facilitate or do not facilitate the decision-making for surgery.

### Implications for policy, practice and research

To the best of our knowledge, our study is the first in assessing uniform criteria for hip replacement surgery that included both surgical patients and patients receiving conservative treatment. We expect the applicability and feasibility of the decision tools to be high. The model without radiology may smoothly find a way in clinical practice since the information needed is easy to obtain. The model may therefore also be applicable in ambulatory settings without a radiologic department. In primary care, the model may for instance be helpful in the decision to refer a patient to secondary care. In future, these kinds of models may also serve as a support tool in primary care for the actual clinical decision to send a patient for surgery. The advantage of a model with radiology is the increased predictive power over a model without radiology. Both tools are meant to serve as an extra tool to facilitate the decision to either perform or not perform surgery and can be used in shared decision-making processes with patients. The tools are not meant to replace the clinical decision-making of the orthopedic surgeon or to rule out the input about preferences from patients themselves.

In the prediction models, we did not include information on social circumstances and patient preferences because the specific aim of this study was to identify uniform clinical criteria to support the surgeon in the decision to perform surgery and to guide the conversation with the patient about treatment options. Therefore, we primarily used objective clinical measures instead of more subjective information such as social circumstances and patient preferences. It goes without saying that relevant information on social circumstances and patient preferences should be considered and added to the result of the decision tool to come to final treatment decisions. In this context, coordination with the primary care setting is crucial as well. Ultimately, not every patient with an indication for hip prosthesis may prefer surgery, whereas others may prefer hip surgery over a prolonged conservative treatment.

The construction of prediction models in clinical decision-making is a promising approach to the improvement of uniform criteria in elective procedures and could serve as a run-up to more objective standards in the future. Besides, improvement in patient-reported outcomes over time, such as delta PROM scores, may further optimize the criteria for the treatment of osteoarthritis patients. Patient profiles should be constructed for those who will benefit most from surgery and those who will benefit most from conservative treatment. Moreover, future research should aim to validate the existing models in another group of patients and surgeons and should also investigate how the surgeons included in the present study, for the blind review, adhere to the novel defined criteria.

## Conclusions

The decision tools will facilitate the decision-making process of physicians on whether to perform hip surgery or not. The models are meant to be guidance in the decision-making with respect to the clinical part. To reach a final treatment decision, information on patient preferences and social context should also be taken into account and added to the outcome of the decision tool.

## Data Availability

The data underlying this article cannot be shared publicly due to privacy reasons of individuals that participated in the study. The data will be shared on reasonable request to the corresponding author.
